# Evaluation of PECAM-1 Gene Polymorphism in Patients with Periodontal Disease and Healthy Individuals

**DOI:** 10.5402/2012/751920

**Published:** 2012-03-05

**Authors:** Mahdi Kdkhodazadeh, Mehrdad Hajilooi, Behzad Houshmand, Sara Khazaei, Leila Gholami, Sara Alijani

**Affiliations:** ^1^Periodontics Department, Dental School, Shahid Beheshti University of Medical Sciences, Hamadan, Iran; ^2^Immunology Department, Dental School, Hamadan University of Medical Sciences, Hamadan, Iran; ^3^Dental School, Hamadan University of Medical Sciences, Hamadan, Iran; ^4^Dental School, Shahid Beheshti University of Medical Sciences, Hamadan, Iran

## Abstract

*Objective.* Our aim in this paper was to investigate the possible genetic association between three Ser563Asn, Leu125Val and Arg670Gly polymorphisms of the PECAM-1 gene and periodontitis. *Methods.* Genomic DNA was isolated from whole blood of 105 periodontal patient (52 with chronic periodontitis and 53 with aggressive periodontitis) and 101 healthy individuals. Samples were genotyped and analyzed for the three single-nucleotide polymorphisms (SNPs) of PECAM-1 using polymerase chain reaction with sequence-specific primers (PCR-SSPs). *Results.* A statistically significant difference was found between the genotypic distribution of the Ser563Asn polymorphism in patients with periodontitis compared to controls (*P* = 0.02). But there were no statistically significant difference between the allele frequencies in the different groups (*P* = 0.05). The other two polymorphisms did not show a statistically significant difference in their allele and genotype frequencies between the groups. There was no statistically significant difference found for any of the polymorphisms allele and genotype distribution in aggressive and chronic periodontitis either. *Conclusions.* No significant association was found between the polymorphism tested and the subgroups of periodontitis, further research is still necessary to determine whether this polymorphism can be used as a genetic marker of periodontitis.

## 1. Introduction

Genetic associations have been claimed to be effective in predisposition of individuals to periodontitis and have been investigated widely in recent years. Periodontal diseases are a result of destructive inflammatory processes affecting the supporting structures of the teeth, causing resorption of alveolar bone, and formation of periodontal pockets [1]. Not everyone is equally susceptible to periodontitis. The risk for development and/or progression of periodontitis are thought to be determined in part by the host's genotype [2]. Recently, there has been increasing interest in identifying allelic variants of genes that can be used in risk assessment of periodontal diseases [3]. Platelet endothelial cell adhesion molecule-1 (PECAM-1/CD31) is a 130-kD vascular cell adhesion and signaling molecule of the immunoglobulin (Ig) superfamily that is expressed on the surface of circulating platelets, monocytes, neutrophils, and selected T-cell subsets. It is also a major constituent of the endothelial cell intercellular junction and plays a role in neutrophil recruitment at inflammatory sites. There is good evidence to suggest that PECAM-1 is a key participant in the adhesion cascade leading to extravasation of leukocytes during the inflammatory process [4]. PECAM-1 is encoded by a 75 kb gene that resides at the end of the long arm of chromosome 17 [5]. It is a relatively large gene (110 kbp) containing 16 exons encoding the 6 Ig-like extracellular domains (exons 3–8), a transmembrane domain (exon 9), and a relatively long cytoplasmic domain (exons 10–16) [6, 7]. Exons 1 and 2 encode the 5′ untranslated regions and the signal peptide [8]. 

Several aminoacid polymorphisms have been identified in PECAM-1. These are located in exon 3 codon 80 altering a valine to a methionine (V80M), exon 3 at codon 125 altering a leucine to a valine (L125V), in exon 8 at codon 563 altering an serine to a asparagine (S563N), and in exon 12 at codon 670 altering an arginine to a glycine (R670G). The latter three polymorphisms are in a strong linkage disequilibrium [9].

These polymorphisms were investigated in the present paper. They have been previously associated with diseases such as ischemic heart diseases, coronary artery disease, stenosis, myocardial infarction (MI), and arthrosclerosis in a number of studies [9–14]. 

Since these diseases are similar to periodontitis, since they all are inflammatory in nature, and no study has yet been undertaken on the possible association between these polymorphisms and periodontal disease, we evaluated the contribution of them in susceptibility to periodontal diseases.

## 2. Material and Methods

In this case, control studies a total of 206 nonsmoking subjects were recruited. 105 patients with periodontitis (36 females and 17 males; range of age: 15–60 years) consisting of 53 patients with aggressive periodontitis (AgP), 52 patients with chronic periodontitis (CP), and 101 healthy individuals. Periodontitis was diagnosed based on of three clinical parameters, probing depth (PD), clinical attachment loss (CAL), and bleeding on probing index (BPI) and radiographic findings. None of patients had a history of current manifestations of systemic diseases. Patients with severe medical disorders (diabetes mellitus, immunological disorders, hepatitis, HIV infection, and cardiovascular involvement), use of orthodontic appliances, needing pre-medication for dental treatment, chronic usage of anti-inflammatory drugs, present acute necrotizing ulcerative gingivitis, and pregnant or lactating woman were excluded from the study.

The diagnostic criteria of CP were the following: (1) the amount of periodontal destruction was consistent with the presence of local factors such as plaque and calculus; (2) at least two sites had probing depth −5 mm and clinical attachment loss −1 mm in every quadrant; the number of the teeth with alveolar bone absorption more than 2/3 root length was less than eight. The clinical criteria of AgP were eight or more teeth, at least 3 of those were other than central incisors or first molars, had a probing depth −5 mm and clinical attachment loss −3 mm. Subjects, even at young ages not reporting familial aggregation of the disease, were not classified as aggressive periodontitis cases and not included in the present paper. In addition, 101 healthy volunteers, without any periodontal disease, systemic inflammatory disease and surgical treatment in the last four weeks were considered as control. The absence of periodontitis was determined according to the following criteria: (1) a minimum of 22 teeth in situ, (2) having no sites with >3 mm probing depth, and (3) the absence of clinical attachment loss. None of the control subjects had a history of periodontitis or tooth loss because of pathogenic tooth mobility. Furthermore, there was no bleeding on probing or radiographic evidence of bone loss in these subjects. All subjects signed an informed consent form before enrolment into the study. 10 mL of venous blood was collected from each subject into tubes containing 50 mM of EDTA. Genomic DNA was isolated from anticoagulated peripheral blood buffy coat using Miller's salting out method [15]. Then the genomic DNA was kept at −80°C until genotyping. The genotyping was performed using PCR-sequence-specific primers (PCR-SSPs) method [16]. Internal control primers were included to control for false negative reactions. The control primers at a 0.2 *μ*M concentration (5′-GCC TTC CCA ACC ATT CCC TT and 5′-TCA CGG ATT TCT GTT GTG TTT C-3′) was used to amplify a 796 bp segment of HLA-DR4 gene. The PECAM-1 Ser563Asn polymorphism was identified by the sequence-specific forward primers and in combination with the consensus reverse primers 5′-CTCTCCCTCCTGTTCCTTGT-3′ and 5′- CGGTGGATGAGGTCCAGATT-3′, respectively. Sequence-specific forward primers in combination with the consensus reverse primers 5′TAGGTCACAATGACGATGTCA-3′ and 5′-GTGGGAAATTATCCACAGTCC-3′, respectively were used to identify the PECAM-1 Arg670Gly polymorphism and the 5′-TGCTGTTCTATAAGGATGACA-3′ forward primer and the 5′-GACTCACCTTCCACCAACAC-3′ common reverse primer were used for investigation of the Leu125Val polymorphism.

Amplification was carried out using a DNA Technology MTC 400 in a total volume of 15 *μ*M that contained 100 ng of genomic DNA, 1 mM each allele-specific primer pair, 200 *μ*M dNTP, 10 mM Tris-HCl (PH = 8.3), 50 mM KCl, 1.5 mM MgCl_2_, and 0.5 IU Taq DNA polymerase. The reaction was carried out as follows; initial denaturation at 94°C for 2 min, followed by 30 cycles of amplification at 96°C for 20 S and annealing at 64°C for 50 S, with extention for 40 S at 72°C, with a final extention for 2 min at 72°C. The amplified PCR products were analyzed by 2% agarose gel electrophoresis followed by 0.5 *μ*g/mL ethidium bromide staining and ultraviolet visualization. The agarose gels were reported by an investigator unaware of the samples.

**Statistics**

Allelic and genotypic frequencies were obtained by direct counting. Contingency tables were used with chi-square tests to compare observed genotype frequencies with those expected under Hardy-Weinberg equilibrium. Chi-square and Fisher's exact tests were used to test for differences in genotype and allele distribution between the groups with the help of SPSS version 13 software. The odds ratio (OR) was calculated along with its 95% CI, and *P* Value < 0.05 was considered statistically significant.The study was approved by the Ethics Committee of University. Written informed consents were obtained before entering the study.

## 3. Results

We investigated the genetic variability of the PECAM-1 gene in 101 healthy controls and 105 patients with a mean age of 33.34 ± 9.8 and 29.12 ± 7.71, respectively.

The results for the Leu125Val and Arg670Gly Allele and genotype frequencies in patients and controls showed no statistically significant difference (Tables 1 and 3).

The allele frequencies of the Ser563Asn polymorphism showed no significant difference between the patients and controls (*P* = 0.05). There was a statistically significant difference in the genotype distribution of this polymorphism. The homozygous genotype combination of Ser/563 was 23.8% in controls, 13.3% in patients (*P* = 0.02) and the homozygous/Asn563 was 4% in controls, 12.4% in patients (*P* = 0.02) (Table 5).

In addition, as shown in Tables 2, 4, and 6, there was no statistically significant difference found in allele and genotype differences between different forms of periodontitis.

## 4. Discussion

Periodontitis is an inflammatory disorder characterized by connective tissue and alveolar bone destruction. The correlation of known polymorphisms in components of the human immune system with phenotypes for certain patient groups currently appears to provide the most promising application of genetic determinants of periodontitis. In this paper we investigated the three most popular SNPs of the PECAM-1 gene. The Ser563Asn polymorphism codes the extracellular domain of the PECAM-1 molecule. We found no significant allele frequency distribution difference between the patients and controls for this polymorphism (*P* = 0.05), but a statistically significant difference in the genotype distribution of this polymorphism was found (*P* = 0.02). Therefore, we cannot totally rule out the association of this polymorphism with periodontitis, but certainly further investigations are needed. Although such polymorphisms usually do not affect the gene expression, it results in an aminoacid change from Ser to Asn in the functional Ig-like domain of PECAM-1, but its role is not yet fully understood. It has been shown that the nutrophil specific antigen (CD177) is involved in transmigration of PMN via their attachment to this domain [17].

Some studies have found relationships between this polymorphism and diseases such as stenosis, myocardial infarction (MI), and arthrosclerosis [10, 11, 13, 14]. But to our knowledge, there have been no similar studies on the association of this polymorphism and periodontitis to compare our results with.

PECAM-1 creates its hemophilic bonds through its first or second Ig-like (d1/2) extracellular domain [18, 19], and these hemophilic bonds have an important role in interendothelial cell bindings [20] and transendothelial migration of monocytes [21, 22].

Since the Leu125Val (C + 373G) polymorphism is in the first and second domain of PECAM-1 and the importance of this domain and also previous findings showing the association of this polymorphism with diseases such as coronary artery disease, atherosclerosis, and ischemic heart diseases; [9, 14] we decided to investigate its possible association with periodontitis.

The results of our paper showed no statistically significant association between allele and genotype frequency in periodontal patients and controls (*P* = 0.61 and *P* = 0.81). We found no similar study to compare our results.

PECAM-1 appears to interact both with it (hemophilic adhesion) and with non-PECAM-1 ligands (heterophilic adhesion).

The cytoplasmic domin of this molecule has an important role in regulating its cellular adhesion behavior and is coded by exons 10–16 [18].

Although the exact role of the exon 12 cytoplasmic domin is not clear yet, the Arg670Gly polymorphism in this exon has been found to be related to diseases such as ischemic heart diseases, MI and Stenosis [10, 12].

Considering the similar immunologic and inflammatory basis of these diseases and periodontitis, we decided to evaluate the possible role of this polymorphism in periodontitis.

However, we found no statistically significant association between the different genotype and allele frequency distribution of this polymorphism and periodontitis.

Taken together, this paper showed no statistically significant relation between these polymorphisms and periodontitis, but further studies are still recommended.

## 5. Conclusion

No significant association was found between the polymorphism tested and the subgroups of periodontitis, further research is still necessary to determine whether this polymorphism can be used as a genetic marker of periodontitis.

## Figures and Tables

**Table 1:** Allele and genotype frequencies of the PECAM-1 and Arg670Gly polymorphism in patients with periodontitis and controls.

PECAM-1 and Arg670Gly polymorphism	Periodontitis *n* = 105	Controls *n* = 101	*P* value
Allele—*n* (%)			
Arg	103 (49%)	99 (48.9%)	0.99
Gly	107 (50.9%)	103 (51%)	
Genotype—*n* (%)			
Gly/Gly	8 (7.6%)	4 (4%)	0.18
Gly/Arg	91 (86.7%)	95 (93.9%)	
Arg/Arg	6 (5.7%)	2 (2%)	

Values are the numbers (percentages) of patients or controls positive for each allele or genotype. The *P* values were calculated by the *χ* ^2^  test from 3 × 2 or 2 × 2 contingency tables for genotypes and alleles, respectively.

**Table 2:** Allele and genotype frequencies of the PECAM-1 and Arg670Gly polymorphisms in aggressive and chronic periodontitis.

PECAM-1 and Arg670Gly					
	Arg/Arg	Gly/Arg	Gly/Gly	Arg	Gly
Aggressive periodontitis	(5.7%)	(88.7%)	(5.7%)	(50%)	(50%)
Chronic periodontitis	(5.8%)	(84.6%)	(9.6%)	(48%)	(51.9%)

No statistical differences were observed at all comparisons (*χ* ^2^  test, *P* > 0.05).

**Table 3:** Allele and genotype frequencies of the PECAM-1 and Leu125Val polymorphism in patients with periodontitis and controls.

PECAM-1 and Arg670Gly polymorphism	Periodontitis *n* = 105	Controls *n* = 101	*P* value
Allele—*n* (%)			
Leu	83 (39.5%)	75 (37.1%)	0.61
Val	127 (60.4%)	127 (62.8%)	
Genotype—*n* (%)			
Leu/Leu	13 (12.4%)	12 (11.9%)	0.81
Leu/Val	57 (54.3%)	51 (50.5%)	
Val/Val	35 (33.3%)	38 (37.6)	

*P* Value <0.05 was considered statistically significant.

**Table 4:** Allele and genotype frequencies of the PECAM-1 and Leu125Val polymorphisms in aggressive and chronic periodontitis.

PECAM-1 and Arg670Gly					
	Leu/Leu	Leu/Val	Val/Val	Leu	Val.
Aggressive periodontitis	(11.3%)	(52.8%)	(35.8%)	(37.7%)	(62.2%)
Chronic periodontitis	(13.5%)	(55.8%)	(30.8%)	(30.7%)	(58.6%)

No statistical differences were observed at all comparisons (*χ* ^2^  test, *P* > 0.05).

**Table 5:** Allele and genotype frequencies of the PECAM-1 and Ser563Asn polymorphism in patients with periodontitis and controls.

PECAM-1 and Ser563Asn polymorphism	Periodontitis *n* = 105	Controls *n* = 101	*P* value
Allele—*n* (%)			
Ser	106 (50.4%)	121 (60%)	0.05
Asn	104 (49.5%)	81 (40%)	
Genotype—*n* (%)			
Ser/Ser	14 (13.3%)	24 (23.8%)	0.02
Ser/Asn	78 (74.3%)	73 (72.3%)	
Asn/Asn	13 (12.4%)	4 (4%)	

*P* Value <0.05 was considered statistically significant.

**Table 6:** Allele and genotype frequencies of the PECAM-1 and Ser563Asn polymorphisms in aggressive and chronic periodontitis.

PECAM-1 and Arg670Gly					
	Ser/Ser	Ser/Asn	Asn/Asn	Ser	Asn
Aggressive periodontitis	(13.2%)	(73.6%)	(13.2%)	(50%)	(50%)
Chronic periodontitis	(13.5%)	(75%)	(11.5%)	(50.9%)	(49%)

No statistical differences were observed at all comparisons (*χ* ^2^  test, *P* > 0.05).
